# Development and validation of the AI attitude scale (AIAS-4): a brief measure of general attitude toward artificial intelligence

**DOI:** 10.3389/fpsyg.2023.1191628

**Published:** 2023-07-24

**Authors:** Simone Grassini

**Affiliations:** ^1^Department of Psychosocial Science, University of Bergen, Bergen, Norway; ^2^Cognitive and Behavioral Neuroscience Lab, University of Stavanger, Stavanger, Norway

**Keywords:** artificial intelligence, questionnaire, factor analysis, psychology, human-computer interaction (HCI)

## Abstract

The rapid advancement of artificial intelligence (AI) has generated an increasing demand for tools that can assess public attitudes toward AI. This study proposes the development and the validation of the AI Attitude Scale (AIAS), a concise self-report instrument designed to evaluate public perceptions of AI technology. The first version of the AIAS that the present manuscript proposes comprises five items, including one reverse-scored item, which aims to gauge individuals’ beliefs about AI’s influence on their lives, careers, and humanity overall. The scale is designed to capture attitudes toward AI, focusing on the perceived utility and potential impact of technology on society and humanity. The psychometric properties of the scale were investigated using diverse samples in two separate studies. An exploratory factor analysis was initially conducted on a preliminary 5-item version of the scale. Such exploratory validation study revealed the need to divide the scale into two factors. While the results demonstrated satisfactory internal consistency for the overall scale and its correlation with related psychometric measures, separate analyses for each factor showed robust internal consistency for Factor 1 but insufficient internal consistency for Factor 2. As a result, a second version of the scale is developed and validated, omitting the item that displayed weak correlation with the remaining items in the questionnaire. The refined final 1-factor, 4-item AIAS demonstrated superior overall internal consistency compared to the initial 5-item scale and the proposed factors. Further confirmatory factor analyses, performed on a different sample of participants, confirmed that the 1-factor model (4-items) of the AIAS exhibited an adequate fit to the data, providing additional evidence for the scale’s structural validity and generalizability across diverse populations. In conclusion, the analyses reported in this article suggest that the developed and validated 4-items AIAS can be a valuable instrument for researchers and professionals working on AI development who seek to understand and study users’ general attitudes toward AI.

## 1. Introduction

Artificial Intelligence (AI) is a terminology that refers to a technology that enables software and machines to emulate human intelligence (see, e.g., Fetzer and Fetzer, 1990). The potential uses of AI systems span various disciplines, including computer science, engineering, biology, neuroscience, and psychology. AI is rapidly transforming various aspects of modern society, including healthcare, transportation, finance, and education (Harari, 2017; Makridakis, 2017). As AI technologies become more integrated into daily life, understanding public attitudes and perceptions toward AI is crucial for guiding their development, adoption, and regulation (Zhang and Dafoe, 2019; Araujo et al., 2020; O’Shaughnessy et al., 2022).

The ongoing progress in AI has led to the development of potentially groundbreaking technological developments such as self-driving cars (Hong et al., 2021). Additionally, AI-based products like Apple’s Siri and Amazon’s Alexa have gained widespread adoption in everyday life (Brill et al., 2019), providing voice-command services for tasks like weather forecasts and navigation. Moreover, AI research has given rise to humanoid robots, such as social robots (Sandoval et al., 2014; Glas et al., 2016; for a review see Hentout et al., 2019). AI’s integration into daily life offers numerous benefits, such as safer driving (Manoharan, 2019), and improved healthcare (Johnson et al., 2021).

This expansion of artificial intelligence presents both exciting opportunities and potential challenges. While AI has the potential to revolutionize numerous industries, it also carries risks such as job displacement due to automation (Tschang and Almirall, 2021). Although AI can generate new employment opportunities, these roles may be markedly different from those that AI replaces (Wilson et al., 2017). Public opinion regarding AI is diverse, with some embracing its benefits, while others exhibit ambivalence or even anxiety (Fast and Horvitz, 2017). Prominent figures such as Stephen Hawking have cautioned that the progression of AI could ultimately endanger human existence (Cellan-Jones, 2014; Kumar and Choudhury, 2022). Similarly, Tesla CEO and tech entrepreneur Elon Musk has expressed concerns about AI development on multiple occasions (Cellan-Jones, 2014). He, along with other experts, has advocated for a ban on military robots (Gibbs, 2017). In March of this year, Musk and several key figures in the AI field penned an open letter calling for a temporary halt to AI development (Pause Giant AI Experiments: An Open Letter, 2023), though this proposal has received mixed reactions from other experts that instead emphasizes as AI can be an opportunity for the future of human kind (Samuel, 2023; see also earlier works as, e.g., Archer, 2021; Samuel, 2021).

Considering the ongoing debate and the numerous perspectives held by various interested parties, stakeholders, and ordinary citizens, it is of paramount importance for psychologists and cognitive scientists interested in human-computer interaction and more in general in the acceptability and adoption of emerging technologies, to develop and implement a concise, reliable, and valid instrument for assessing attitudes toward AI for assessing attitudes toward AI, that can be easily implemented and assessed. Investigating this area can yield invaluable insights into the determinants influencing individuals’ acceptance or resistance to AI, as well as the potential benefits and risks they associate with its deployment (Nadimpalli, 2017; Eitel-Porter, 2021). Moreover, understanding these factors can aid in the development of educational initiatives and public awareness campaigns that address concerns and misconceptions about AI while emphasizing its potential for positive impact and rationally inform about possible dangers. By fostering a deeper understanding of public sentiment and expectations surrounding AI, researchers and policymakers can work together to ensure that AI is developed and implemented responsibly and ethically. Such collaboration will serve to maximize the benefits of AI while mitigating its potential risks, ultimately leading to a more harmonious integration of AI technologies into our daily lives and society.

To develop the scale items, recent literature on the topic of AI perception and attitude was examined to identify major themes and previous efforts to create similar scales (see, e.g., Schepman and Rodway, 2020; Sindermann et al., 2021). It was found that experts, the public, and the media all expressed both positive and negative views about AI (Fast and Horvitz, 2017; Anderson et al., 2018; Cave et al., 2019). Large-scale surveys confirmed these mixed perspectives and reflected similar key themes (Zhang and Dafoe, 2019). Public concerns included job displacement, ethical issues, and non-transparent decision-making. In contrast, positive attitudes centered on AI’s potential to improve efficiency and provide innovative solutions in various domains. Interestingly, a phenomenon called “algorithm appreciation” has been reported, wherein AI was preferred over humans in certain contexts (Logg et al., 2019).

Some studies delved deeper into specific aspects of attitude toward AI. For instance, potential job loss emerged as a significant concern due to the high computerizability of numerous occupations (Chui et al., 2016; Frey and Osborne, 2017). Ethical concerns were also prominent, particularly in medical settings (Morley et al., 2020). Public opinion was divided on using AI for tasks traditionally performed by medical staff, and many people felt uncomfortable with personal medical information being used in AI applications. Vayena et al. (2018) suggested that trust in AI could be promoted through employing measures for personal data protection, unbiased decision-making, appropriate regulation, and transparency, a view supported by other recent discussions on trust in AI (Schaefer et al., 2016; Barnes et al., 2019; Sheridan, 2019; Middleton et al., 2022).

Although there are existing instruments that evaluate people’s acceptance of technology (e.g., Davis, 1989; Parasuraman and Colby, 2015), most of them do not specifically address AI, which may differ in key aspects regarding acceptance. The Technology Acceptance construct (Davis, 1989) primarily emphasizes a user’s willingness to adopt technology through consumer choice. However, AI adoption frequently does not involve consumer choice, as large organizations and governments often implement AI without seeking input from end users, who are then left with no option but to engage with it. Consequently, traditional technology acceptance measures may not be ideally suited for gauging attitudes toward AI. Other existing measures tend to be lengthy, context-specific, or lack empirical validation. Additionally, most questionnaires designed for evaluating technological attitudes and acceptance (e.g., The Media and Technology Usage and Attitudes Scale - MTUAS, see Rosen et al., 2013) have not been specifically developed and validated considering modern consumer-oriented AI systems, such as OpenAI ChatGPT or text-to-image AI services.

In recent years, few scales have been developed specifically with the aim to assess attitudes toward AI. Schepman and Rodway (2020) introduced the General Attitudes toward Artificial Intelligence Scale (GAISS), a 20-item scale with a two-factor structure (positive and negative attitudes toward AI). Sindermann et al. (2021) proposed a concise 5-item scale, the Attitude Toward Artificial Intelligence scale (ATAI), featuring a two-factor structure (acceptance and fear). Another scale (Threat of Artificial Intelligent Scale, TAI; Kieslich et al., 2021) has also been developed to specifically assess fear in AI technology. However, these scales face challenges concerning their practicality and possible use in the context of assessing general attitude.

The GAISS, with its many items, can be time-consuming to administer, making it less suitable for large-scale surveys or situations with limited participant time. In contrast, the ATAI scale is easier to administer but emphasizes extreme outcomes with items like “Artificial intelligence will destroy humankind” or “Artificial intelligence will benefit humankind.” This approach may not capture the nuanced attitudes people may have, which could include a mix of optimism, skepticism, and concerns about specific issues. The scale also appears to focus more on negative AI aspects (fear, destruction, and job losses) than on positive or factual aspects, potentially leading to an overemphasis on negative attitudes and a less accurate representation of people’s overall AI perceptions. The ATAI scale also highlights emotions, with three out of five items loading on the “fear” factor and only two on the “acceptance” factor in the two-factor model proposed by Sindermann et al. (2021).

Moreover, both scales were developed and tested in a context where large language models (LLMs) like ChatGPT were not yet widely available and the public debates following these AI systems did not emerge yet (for a review see Leiter et al., 2023) or discussed. The rapid implementation of these AI systems and increased exposure to information about them may have significantly altered public attitudes toward AI technology and how these systems are perceived as useful and impactful for the future. Developing a concise, reliable, and valid scale for assessing AI attitudes would facilitate research and practice in understanding and addressing public perceptions of AI. A short and usable scale would also support non-psychologists, as AI professionals and the technology sector in gathering critical individual variables when testing products for the general population.

### 1.1. The present study

This study aims to address the existing gap in scientific understanding by creating and validating the AI Attitude Scale (AIAS), a concise instrument designed to assess public attitudes toward AI. The AIAS strives to be a brief yet dependable tool that is easy and quick to administer for both psychological research and non-psychologists interested in gauging users’ or citizens’ attitudes toward AI, such as software developers, businesses, organizations, and stakeholders.

Drawing on established theoretical frameworks, such as the Technology Acceptance Model (TAM) (Davis, 1989) and the Unified Theory of Acceptance and Use of Technology (UTAUT) (Venkatesh et al., 2003), as well as empirical research on AI-related risks (Cheatham et al., 2019; Kaur et al., 2022), and societal implications (Ernst et al., 2019; Tschang and Almirall, 2021). The scale considers various aspects of AI’s potential influence on individuals’ lives, work, and the broader human experience, while also addressing the likelihood of future AI adoption. The AIAS scale is designed to capture attitudes toward AI by focusing on the perceived utility and potential impact of technology on society and humanity, including perceived benefits, potential risks, and intentions to use AI technologies. In line with items (and theoretical standpoints) discussed in similar scales (GAISS and ATAI), the AIAS also includes some items related that can be related to emotional statements, however, does not focus in evaluating AI-elicited emotions but attempts to provide a balanced assessment of attitudes toward AI.

The present article, based on 2 studies from distinct populations, outlines the development and validation of the AIAS, starting with an examination of the theoretical underpinnings of the scale items and the methods employed in scale development. Subsequently, the results of an exploratory factor analysis (EFA) and an assessment of the scale’s internal consistency and convergent validity are presented. Finally, the scale factor structure was evaluated using confirmatory factor analysis (CFA) with a different sample of participants.

## 2. Methods

### 2.1. Scale development

The initial AIAS consisted of 5 items. One of the items was reverse scored to reduce response bias (DeVellis and Thorpe, 2021). Participants rated their agreement with each item using a 10-point Likert scale (1 = Not at all, 10 = Completely Agree). A 10-point scale was chosen for the high test–retest reliability, easy to use (Preston and Colman, 2000), as well as good level of granularity. The selection of these specific items was based on several considerations. Primarily, the intent was to provide a comprehensive coverage of key dimensions of attitudes toward AI, derived from prior theoretical and empirical work in technology acceptance and risk perception literature. This included factors such as perceived usefulness, potential societal impact, adoption intention, risk perception, and overall evaluation of AI’s impact on humanity. Furthermore, it was sought to incorporate the breadth of public opinion, acknowledging that attitudes toward AI can range from optimistic enthusiasm to concern and even fear of potential risks. The items originally included in the t-items scale were the following:

1) I believe that AI will improve my life.

This item was derived from the Technology Acceptance Model (TAM) (Davis, 1989), which posits that perceived usefulness (i.e., the degree to which an individual believes that using a technology will enhance their performance) is a primary determinant of technology acceptance. In the context of AI, this item assesses individuals’ beliefs about the potential benefits of AI in improving their quality of life. Such theory has been applied recently in the field of AI (see, e.g., Sohn and Kwon, 2020).

2) I believe that AI will improve my work.

Like the first item, this item is also grounded in the TAM (Davis, 1989) and reflects individuals’ beliefs about the potential benefits of AI in the work domain. This item addresses the role of AI in generally improving human work condition, in line with expectations of AI technology discussed in the current scientific literature (Wijayati et al., 2022).

3) I think I will use AI technology in the future.

This item is based on the concept of behavioral intention, which is central to several theories of technology acceptance, including the TAM (Davis, 1989) and the Unified Theory of Acceptance and Use of Technology (UTAUT) (Venkatesh et al., 2003), including its current discussion in relationship with AI (Venkatesh, 2022). Behavioral intention is considered a proximal determinant of actual technology use, and this item measures individuals’ intentions to adopt and use AI technologies in the future.

4) I think AI technology is a threat to humans (reverse item).

This reverse-scored item assesses individuals’ concerns about the potential risks and negative consequences of AI technology. The item is grounded in the literature on the risk of super intelligent AI (Barrett and Baum, 2017) and risk perception associated with technology (Slovic, 1987; Frey and Osborne, 2017). These theoretical standpoints highlight the importance of understanding individuals’ concerns about the potential dangers posed by AI, such as job displacement, loss of privacy, or even existential threats.

5) I think AI technology is positive for humanity.

This item captures individuals’ overall evaluation of the impact of AI technology on society and humanity as a whole. The item is grounded in the literature on the social implications of AI (Brynjolfsson and McAfee, 2014) and reflects the belief that AI can contribute to societal progress and well-being by addressing complex global challenges, improving healthcare (Loh et al., 2022), enhancing education (Xia et al., 2022; Yang, 2022), and fostering economic growth (Jones, 2022; Matytsin et al., 2023).

In conclusion, the selection of these items was aimed at achieving a balance between theoretical grounding, and coverage of the range of public attitudes toward AI. It is also noteworthy that these items may not be exhaustive and cover all the aspects related to AI attitude, and that future iterations of the scale may incorporate additional items to further enrich the measurement of attitudes toward AI.

### 2.2. Participants and procedure

Prior to the experiments, participants were given an overview on what it is AI, and how it is used. This included information on current AI developments like virtual assistants, content recommendation algorithms for media streaming services, and AI-powered communication tools such as grammar checkers and chatbots. These uses of AI systems were explicitly mentioned in the information document prior to the presentation of the questionnaires. The aim was to engage the participants in thinking about the essence of AI and its ongoing advancements, thus minimizing potential sources of misinterpretation.

In the first study (EFA), a gender-balanced convenience sample of 230 UK adults was assembled using Prolific, an online platform for participant recruitment. A sample size of >200 for factor analysis is generally accepted as fair according with the literature (Comrey and Lee, 2013). All participants indicated they used a computer or laptop with a physical keyboard. The sample size was determined by the number of variables analyzed in a more comprehensive survey on AI-generated data perception, including the questions investigated in this article. EFA is typically used for exploratory purposes, where the goal is to uncover the underlying factor structure of a set of observed variables, and it is a data-driven technique that allows for flexibility in model specification. While the scale items emerged from theoretical standpoints, it was preferred to first establish their underlying factor structure through EFA to allow for an unbiased exploration of the data, and secondly perform a CFA with a different participant sample.

For the second study (CFA), a separate convenience sample of 300 US adults, with characteristics like the UK sample, was recruited through the same platform. The sample size was based on the number of variables examined in an extensive survey focusing on human-computer and human-AI interaction, incorporating the questions examined in this article.

In both studies, participants were required to be fluent in English and over 18 years old. All participants in both studies were asked to read and explicitly accept an informed consent form before participating. They were informed about the tasks they would be performing and reminded of their right to withdraw from the study at any time. Both studies adhered to the Declaration of Helsinki for scientific studies involving human participants and complied with local and national regulations. No personally identifiable information was collected from study participants.

Participants in both studies completed an online survey that included the questions included in the AIAS, along with other measures of attitudes toward technology, as well as demographic questions. The attitude dimension of the Media and Technology Usage and Attitudes Scale (MTUAS, Rosen et al., 2013), was measured and used to explore convergent validity in both studies. The MTUAS is a comprehensive psychometric instrument designed to assess individuals’ usage patterns and attitudes toward various forms of media and technology. It has shown to have a good validity as psychometric measure (e.g., Sigerson and Cheng, 2018). This scale encompasses multiple dimensions, and the one considered in the present study are positive and negative attitudes toward technology, and anxiety related to technology use. The MTUAS has been extensively used by researchers and practitioners to gain insights into how people interact with and perceive technology in their daily lives or in professional environments (e.g., Rashid and Asghar, 2016; Becker et al., 2022; Srivastava et al., 2022). The MTUAS factors of positive, negative, multi-tasking and anxiety were included in the original survey (as they are presented together in the original scale), however, after preliminary analyses it was decided to not report the analyses for the multi-tasking factor of the scale, as not directly related to technology attitude.

For both studies, the surveys were developed using Psyktoolkit (Stoet, 2010, 2017). The surveys included a questionnaire battery containing attention check questions, such as “select the highest value for this item” or “select the lowest value for this item.” Participants who failed one or more attention checks were excluded from the sample and replaced until the pre-determined final samples were reached. In the first study, six participants needed replacement, while in the second study, 18 participants required replacement due to failing one or more attention checks.

In the survey, the sample characteristics were examined, including age, gender, and level of education. In study 1, the sample featured a balanced distribution of males and females (114 each), with two participants choosing not to answer the question or selecting a third gender option. The ages of the participants ranged from 18 to 76 years old (*M* = 40.2, *SD* = 14.6). A majority held a university degree (59.6%), followed by high school (38.7%). A smaller portion of the sample had completed middle school (0.9%) or elementary school (0.9%), and none reported having no formal schooling. The age of the participants ranged from 18 to 76 years old (*M* = 41.5, *SD* = 15). A majority held a university degree (60.7%), followed by high school (38.7%). A smaller portion of the sample had completed middle school (0.9%) or elementary school (0.9%), and none reported having no formal schooling.

In study 2, the sample as well featured a balanced distribution of males (*N* = 142) and females (*N* = 150), with eight participants choosing not to answer the question or selecting a third gender option. The ages of the participants ranged from 18 to 81 years old (*M* = 40.2, *SD* = 14.6). A majority held a university degree (59.6%), followed by high school (39%), and only one reported only completion of middle school (0.3%). None of the participants reported having completed only elementary school or not having any formal schooling. The age of the participants ranged from 18 to 76 years old (*M* = 40.2, *SD* = 14.6). A majority held a university degree (59.6%), followed by high school (38.7%). A smaller portion of the sample had completed middle school (0.9%) or elementary school (0.9%), and none reported having no formal schooling.

### 2.3. Data analysis

EFAs were performed in data from Study 1 to assess the factor structure of the AIAS (Fabrigar and Wegener, 2011). The factor structure (1 factors, 4 items) identified in study 1, was further validated using a CFA with the data collected in study 2. Internal consistency, and convergent validity with related measures were also examined for the sample in both studies (Cronbach, 1951; Shrout and Fleiss, 1979). Correlation matrixes were computed to evaluate convergent validity of the developed scale with the MTUAS scale (positive, negative, and anxiety factors). Multiple linear regressions were computed to understand the relationship between the background information collected for the samples and AIAS score. Data analysis was performed using the statistical software Jamovi 2.3.21 (The Jamovi Project, 2022).

## 3. Results

### 3.1. Study 1 (EFA)

#### 3.1.1. The 5-items original scale

First, descriptive statistics were obtained for each item of the scale. The descriptive statistics for the AI attitude scale items are presented in Table 1. The skewness values for the items indicated that the data is roughly symmetric. The kurtosis values for the items indicated that the data is platykurtic.

**Table 1:** Descriptive statistics for each item.

	Items				
	1	2	3	4	5
Mean	5.06	5.09	6.19	5.82	5.57
Median	5	5	6	6	5
Standard deviation	2.27	2.42	2.33	2.46	1.99
Skewness	−0.114	−0.0612	−0.322	−0.0469	0.0992
Std. error skewness	0.16	0.16	0.16	0.16	0.16
Kurtosis	−0.622	−0.686	−0.364	−0.78	−0.0172
Std. error kurtosis	0.32	0.32	0.32	0.32	0.32

The data was analyzed to assess whether the assumptions necessary for performing an EFA were satisfied. The Kaiser-Meyer-Olkin (KMO) measure of sampling adequacy, a critical index for determining the suitability of the data for factor analysis, was computed. With an overall KMO value of 0.827, the data demonstrated meritorious sampling adequacy, thereby confirming its appropriateness for exploratory factor analysis (Williams et al., 2010).

Furthermore, Bartlett’s test of sphericity was conducted to evaluate the presence of significant correlations between the items, a prerequisite for factor analysis. The test yielded a significant result [χ^2^(10) = 612, *p* < 0.001], providing evidence of substantial correlations among the items. Consequently, these findings support the validity of proceeding with the EFA.

In the EFA, maximum likelihood extraction method and oblimin rotation were used. Oblimin rotation was preferred as it was assumed that the factors extracted may correlated with each other. Two factors were extracted based on parallel analysis.

As shown in Table 2, the factor loadings suggest that Items 1, 2, and 3 loaded moderately to highly on Factor 1, while Items 4 and 5 loaded highly on Factor 2. The uniqueness values indicate the proportion of variance in each item that is not explained by the factors. Inter-factor correlation was shown to be high (0.749), confirming the adequacy of the use of Oblimin rotation method. Reliability of the scale was analyzed using Cronbach alpha and McDonald’s omega.

**Table 2:** Factor loadings from the EFA of the 5 items scale.

	Factor		
	1	2	Uniqueness
Item 1	0.731		0.236
Item 2	0.965		0.189
Item 3	0.680		0.338
Item 4		0.513	0.848
Item 5		0.865	0.125

“Maximum likelihood” extraction method was used in combination with an “oblimin” rotation.

Table 3 shows the mean scores, standard deviations, Cronbach’s alpha, and McDonald’s omega values for each factor and for the entire scale. Factor 1, with a Cronbach’s alpha of 0.892 and McDonald’s omega of 0.892, showed good internal consistency. Factor 2 with a Cronbach’s alpha of 0.496 and McDonald’s omega of 0.504, showed an overall internal consistency lower than Factor 1. The entire scale had a Cronbach’s alpha of 0.830 and McDonald’s omega of 0.860, indicating good internal consistency.

**Table 3:** Mean, SD, Cronbach’s alpha, and McDonald’s omega values for each factor and for the entire scale.

	Mean	*SD*	Cronbach’s α	McDonald’s ω
Factor 1	5.45	2.12	0.892	0.892
Factor 2	5.69	1.82	0.496	0.504
Full scale	5.54	1.78	0.830	0.860

A correlation matrix was computed to visualize the correlations between the questionnaire items. Table 4 shows a matrix of the 5 items included in the AI attitude scale.

**Table 4:** Correlation between the items included in the AIAS questionnaire.

	Correlation matrix								Item 1	Item 2	Item 3	Item 4	Item 5
Item 1	Pearson’s r	–					*p*-value	–				
Item 2	Pearson’s r	0.772***	–				*p*-value	< 0.001	–			
Item 3	Pearson’s r	0.711***	0.716***	–			*p*-value	< 0.001	< 0.001	–		
Item 4	Pearson’s r	0.225***	0.153*	0.167*	–		*p*-value	< 0.001	0.02	0.011	–	
Item 5	Pearson’s r	0.707***	0.63***	0.662***	0.337***	–	*p*-value	< 0.001	< 0.001	< 0.001	< 0.001	–

Significant correlations are marked.

*p < 0.05, ***p < 0.001.

These analyses showed relatively poor loading of item 4 in the established factors. Therefore, the item was removed from the analyses and the scale analyzed again without the item.

#### 3.1.2. The 4-items revised AIAS scale

Due to the relatively poor loading on factor 2 and the poor correlation with the other items, item 4 of the questionnaire was eliminated from the analyses, trying to establish a scale with better psychometric characteristics compared to the 2-factors structure previously proposed. For the 4-items questionnaire, the Kaiser-Meyer-Olkin (KMO) measure of sampling adequacy obtained a value of 0.83, demonstrated again meritorious sampling adequacies (Williams et al., 2010). Bartlett’s test of sphericity yielded a significant result [χ^2^(6) = 583, *p* < 0.001], providing evidence of substantial correlations among the items. For the EFA of the 4-items version of the questionnaire were sed the same extraction and rotation methods as the ones previously used (maximum likelihood and oblimin rotation). Table 5 shows that all the items highly load on just one factor.

**Table 5:** Factor loadings from the EFA of the 4 items scale.

	Factor	
	1	Uniqueness
Item 1	0.892	0.204
Item 2	0.856	0.267
Item 3	0.821	0.327
Item 5	0.778	0.394

“Maximum likelihood” extraction method was used in combination with “oblimin” rotation.

The 4-items scale shows a mean score of 5.48 (*SD* = 1.99) and a Cronbach’s alpha of 0.902 and McDonald’s omega of 0.904, indicating good internal consistency, and exceeding the internal consistency reported for both the total 5-items scale and the 3-items factor 1 identified in the previous analyses.

Correlation analyses were computed between the 4-items AIAS and the attitude factors of the Media and Technology Usage and Attitudes Scale (MTUAS), as previously. The correlation matrix is presented in Table 6. The analyses for the 4-items AIAS do not show significant different relationship with the attitude dimensions of MTUAS compared to what observed for the same analyses where the Factor 1 of the 2-factors 5-items AIAS was included in the analyses, confirming the interpretation of the relationship between AIAS and MTUAS proposed earlier.

**Table 6:** Correlation matrix for the factors of MTUAS-attitude and the 1-factors 4-items AIAS scale.

		MTUAS-p	MTUAS-n	MTUAS-a	4-items AIAS
MTUAS-p	Pearson’s r	–				*p*-value	–			
MTUAS-n	Pearson’s r	−0.323***	–			*p*-value	< 0.001	–		
MTUAS-a	Pearson’s r	0.513***	−0.124	–		*p*-value	< 0.001	0.061	–	
4-items AIAS	Pearson’s r	0.484***	−0.301***	0.245***	–	*p*-value	< 0.001	< 0.001	< 0.001	–

Significant correlations are marked.

***p < 0.001.

A multiple linear regression was computed to understand how the background information collected for the sample can explain the total score of the 4-items AIAS. The analysis was conducted to examine the relationships between age, gender, and education levels with the total score of the 4-items AIAS. The overall model was statistically significant.

Gender emerged as the only statistically significant predictors of the 4-items AIAS total score among the ones used in the model, with female participants showing lower scores compared to male participants. Age and education levels (*p* > 0.05 for all comparisons) were not found to be significant predictors of the 4-items AIAS total score. Table 7 reports the results of the multiple linear regression model. Estimates are obtained with ordinary least squares minimizing the sum of squared deviations between each observed value and predicted values.

**Table 7:** Multiple linear regression model summarizing the relationships between age, gender, and education levels as predictors of 4-items AIAS total score.

	Predictor	Estimate	SE	*p*
Interceptᵃ	6.21712	0.41659	14.924	< 0.001
Age	−0.00872	0.00879	−0.992	0.322
Gender				
Female–Male	−0.78393	0.25834	−3.035	0.003
Education				
HS—U	0.11284	0.26564	0.425	0.671
MS—U	−1.86291	1.37828	−1.352	0.178
ES—U	0.80761	1.38322	0.584	0.560

^a^Represents reference level. U, University; HS, High School; MS, Middle School; ES, Elementary School.

### 3.2. Study 2 (CFA)

In study 2, the 4-items version of the AIAS validated from study 1 was tested, and CFA was conducted. Descriptive statistics were calculated for each of the four items in the scale. Table 8 presents the descriptive statistics for the AI attitude scale items.

**Table 8:** Descriptive statistics for each item of 4-items AIAS in study 2.

	Items			
	1	2	3	4
Mean	5.8	5.53	7.14	6.17
Median	6	5	7	6
Standard deviation	2.25	2.54	2.36	2.2
Skewness	−0.131	−0.0571	−0.552	−0.271
Std. error skewness	0.141	0.141	0.141	0.141
Kurtosis	−0.435	−0.758	−0.368	−0.234
Std. error kurtosis	0.281	0.281	0.281	0.281

A Confirmatory Factor Analysis was conducted to examine the factor structure of the 4-item questionnaire with the sample from Study 2. The factor loadings for all items were statistically significant, suggesting that the items were strong indicators of the underlying factor. Results including standardized estimate (Rosseel, 2012) are reported in Table 9.

**Table 9:** CFA factor loading and statistics including standard estimate.

Factor loadings						
Factor	Indicator	Estimate	SE	*Z*	*p*	Stand. estimate
Factor 1	Item 1	2.11	0.100	21.1	< 0.001	0.941
	Item 2	2.12	0.121	17.6	< 0.001	0.838
	Item 3	1.78	0.118	15.1	< 0.001	0.757
	Item 4	1.84	0.105	17.6	< 0.001	0.839

The model fit was assessed using several fit indices. The chi-square test for exact fit was not significant, indicating a good model fit [χ^2^(2) = 2.49, *p* = 0.289] (but see DiStefano and Hess, 2005). Additionally, the Comparative Fit Index (CFI) and the Tucker-Lewis Index (TLI) were both above the recommended threshold (see Byrne, 2016) of 0.95 (CFI = 0.999, TLI = 0.998), suggesting a good fit between the hypothesized model and the observed data. The results for the Root Mean Square Error of Approximation (RMSEA) indicate a good model fit as the value is below the suggested cutoff (MacCallum et al., 1996) of 0.05 (RMSEA = 0.0285, 90% CI [0 0.122]). Overall, the Confirmatory Factor Analysis results support the proposed single-factor structure of the 4-item questionnaire, with strong factor loadings and a good model fit (see guidelines as in Sun, 2005). Reliability analysis was then computed similarly as in Study 1. The analysis for the 4-items scale indicated good internal consistency, similar as what was observed for Study 1. A correlation table was computed to visualize the correlations between the questionnaire items. Table 10 shows a correlation matrix of the 4 items included in the second version of the AI attitude scale.

**Table 10:** Correlation between the items included in the AIAS questionnaire.

		Item 1	Item 2	Item 3	Item 4
Item 1	Pearson’s r	–				*p*-value	–			
Item 2	Pearson’s r	0.793***	–			*p*-value	< 0.001	–		
Item 3	Pearson’s r	0.704***	0.637***	–		*p*-value	< 0.001	< 0.001	–	
Item 4	Pearson’s r	0.79***	0.687***	656***	–	*p*-value	< 0.001	< 0.001	< 0.001	–

Significant correlations are marked.

***p < 0.001.

Similarly, to what was done for Study 1, an additional statistical analysis was conducted to assess the convergent validity between the Artificial Intelligence Attitude Scale (AIAS) and the attitude components of the Media and Technology Usage and Attitudes Scale (MTUAS). Pearson’s correlation coefficients were calculated between the attitude components of MTUAS and the two-factor components of AIAS. The correlation matrix is shown in Table 11. The results of the correlation matrix confirm the results already reported for the 4-items AIAS shown in Study 1, with the AIAS being moderately associated with MTUAS-p and MTUAS-n, and weakly associated with MTUAS-a. Results are shown in Table 12.

**Table 11:** Correlation matrix for the factors of MTUAS-attitude and the 4-items AIAS scale.

		MTUAS-p	MTUAS-n	MTUAS-a	4-items AIAS
MTUAS-p	Pearson’s r	–				*p*-value	–			
MTUAS-n	Pearson’s r	−0.272	–			*p*-value	< 0.001	–		
MTUAS-a	Pearson’s r	0.330	−0.100	–		*p*-value	< 0.001	0.084	–	
4-items AIAS	Pearson’s r	0.535	−0.353	0.144	–	*p*-value	< 0.001	< 0.001	0.013	–

**Table 12:** Multiple linear regression model summarizing the relationships between age, gender, and education levels as predictors of 4-items AIAS total score for Study 2.

Predictor	Estimate	SE	*t*	*p*
Intercept^a^	7.1230	0.37587	18.9504	< 0.001
Age	−0.0100	0.00793	−1.2636	0.207
Gender				
Female–Male	−0.8601	0.23887	−3.6008	< 0.001
Education				
HS – U	0.0373	0.78748	0.0473	0.962
MS– U	−0.2759	0.24057	−1.1467	0.252
ES – U	−2.3997	2.16407	−1.1089	0.268

ᵃRepresents reference level. U, University; HS, High School; MS, Middle School; ES, Elementary School.

A further multiple linear regression was computer to understand how the background information collected for the sample can explain the total score of the 4-items AIAS for the sample of Study 2. These analyses follow the same steps of the multiple linear regression performed for the sample of Study 1. The overall model was statistically significant.

As for the analysis reported for Study 1, gender emerged as the only statistically significant predictors of the 4-items AIAS score among the ones used in the model, with female participants showing lower scores compared to male participants. Age and education levels were not found to be significant predictors of the 4-items AIAS total score. Table 12 reports the results of the multiple linear regression model. Estimates are obtained as described earlier.

## 4. Discussion

In this article, the AI Attitude Scale (AIAS) is presented, and its psychometric properties were evaluated. The AIAS items were developed based on established theoretical frameworks and empirical research in technology acceptance, AI, and risk perception (Davis, 1989; Venkatesh et al., 2003; Barrett and Baum, 2017). The scale captures key dimensions of AI attitudes, including perceived benefits, potential risks, and intentions to use AI technologies (Brynjolfsson and McAfee, 2014; Bostrom, 2016). Drawing from the Technology Acceptance Model (TAM), the Unified Theory of Acceptance and Use of Technology (UTAUT), and literature on AI-related risks and societal implications, the AIAS provides a quick and psychometrically validated approach to understanding general attitude toward AI (e.g., dos Santos et al., 2019; Persson et al., 2021; Scott et al., 2021; Vasiljeva et al., 2021; Young et al., 2021). Furthermore, some of the items included in the AIAS were similar to items that were included in scales previously developed to understand attitude toward AI: Item 4 of the AIAS preliminary 5-items scale is similar with the item number 3 of the ATAI scale (“Artificial intelligence will destroy humankind”), however, it has a softer and more nuanced tone, not directly suggesting that AI will annihilate humankind. Item 5 of the preliminary version of the scale is also similar with the item number 4 of the ATAI scale (“Artificial intelligence will benefit humankind”).

An exploratory factor analysis (EFA) was conducted to investigate the factor structure of the original 5-item AIAS scale. The preliminary EFA indicated that the scale could be divided into two factors with moderate-to-high correlations between them. Factor 1, consisting of items related to individual attitudes toward AI (AIAS-IA), comprises the following items: “I believe that AI will improve my life,” “I believe that AI will improve my work,” and “I think I will use AI technology in the future.” Factor 2, which can be interpreted as a social attitude toward AI and its risks, includes the items “I think AI technology is a threat to humans” and “I think AI technology is positive for humanity.” Factor 1 demonstrated high internal consistency and reliability, as evidenced by the high values of Cronbach’s alpha coefficient and McDonald’s omega coefficient (>0.8) (Venkatesh, 2022). This suggests that the items in Factor 1 are closely related and measure the same underlying construct of attitudes toward the potential benefits of AI. The high reliability of Factor 1 indicates that it is a suitable and trustworthy measure for assessing attitudes toward the positive aspects of AI. In contrast, Factor 2 exhibited lower internal consistency and reliability compared to Factor 1, with a Cronbach’s alpha coefficient value of 0.496 and a McDonald’s omega coefficient value of 0.504 (Rosen et al., 2013). These findings imply that the items in Factor 2 may not be as strongly related or measure the same underlying construct as effectively as Factor 1. While Factor 2 may still be useful in assessing attitudes toward the potential risks of AI, further development of this factor may be needed in future studies.

The 4-items AIAS scale, developed by excluding item 4 from the original 5-item scale, demonstrated higher levels of internal consistency compared to the previously analyzed 2-factor scale. This suggests that the 4-item scale may provide a more reliable and cohesive measure of AI attitudes, thereby enhancing the usefulness of the AIAS in research and future applications within the information systems domain (Collins et al., 2021). The excluded item 4 may have been poorly understood by participants or might have measured a different psychological construct. Upon examination, this item could potentially be measuring fear toward AI (or a perceived direct threat posed by AI technology), rather than general positive or negative attitudes toward the technology (this is in line with the fact that such item is reported in the “fear” factor in the Sindermann et al., 2021).

In the survey, sample background variables were tested as predictors of the 4-items AIAS total score. Among the independent variables, gender emerged as the only statistically significant predictor of the 4-items AIAS total score. Female participants scored lower compared to males, suggesting that men may have more positive attitudes toward AI technology. This finding aligns with existing research indicating that men generally have more positive attitudes and higher levels of acceptance toward technology than women (e.g., Schumacher and Morahan-Martin, 2001; Ong and Lai, 2006; Ziefle and Wilkowska, 2010). However, the effect could depend on the specific technology (e.g., da Silva and Moura, 2020).

A confirmatory factor analysis (CFA) was conducted in a second study with 300 participants from the USA, who had similar characteristics to the first study’s sample. The analysis confirmed that the 1-factor model (4-items) of the IAPS demonstrated an adequate fit to the data, providing further evidence for the scale’s structural validity and generalizability across different populations. Follow-up analyses in Study 2 confirmed that the AIAS exhibited a good level of internal consistency, comparable to the findings in Study 1. Additionally, further analyses on convergent validity (with the analyzed factors of MTUAS) and individual differences of participants revealed similar trends to those reported in Study 1, strengthening the generalizability of the reported findings.

In summary, the reported analyses suggest the use of the one-factor 4-items scale. Future studies may want to further investigate how to efficiently measure social attitude toward AI, reworking and modifying items n. 4 and n. 5 from the originally proposed 5-items AIAS scale, or developing multi-factorial scales that specialized in particular aspects of the attitude toward AI technology.

### 4.1. Limitation and future research

The present research has some limitations that should be considered by researchers and professionals interested in using the proposed scale. First, the sample used in this study is not representative of the general population. The samples should be considered a convenience sample from UK (study 1) and USA (study 2) population, and the findings may not generalize to other populations with different demographic characteristics or cultural backgrounds (Bryman, 2016). As the sample was recruited online among people offering to perform research tasks using the online platform Prolific, the sample may be composed of people who are generally more IT-savvy compared to the general population. Despite participants using Prolific having generally been found to deliver high data quality (see, e.g., Peer et al., 2017), participants in online studies can be less motivated compared to, e.g., students participating in filling surveys in campus. However, the uses of control question for attention check, should have limited participants (or excluded those) that responded randomly or without reading all the items. Future research could validate the AIAS in more diverse samples, including those from different countries and selected age groups, to enhance the scale external validity and its possible impact.

Furthermore, this study only utilized self-report measures, which may be subject to social desirability bias or response biases (Podsakoff et al., 2003). Future research could benefit from incorporating behavioral, physiological, and implicit measures to complement self-report data, enhancing the understanding of AI attitudes as both a phenomenon and a psychological construct (Rosen et al., 2013). Physiological measures, in particular, could be employed in lab-based experiments where participants are exposed to information about AI or engage with AI directly. These measures may help researchers to better comprehend automatic reactions to AI-related stress or negative dispositions toward the technology.

For example, researchers could track heart rate variability, skin conductance, and cortisol levels as indicators of stress responses or arousal when individuals interact with AI technologies or are presented with AI-related stimuli. Similarly, facial expressions or eye-tracking data could provide valuable information on attention, cognitive processing, and emotional responses to AI. By incorporating these physiological measures, researchers can gain a more comprehensive and nuanced understanding of the factors influencing individuals’ attitudes toward AI and their subsequent behavior (as has been done in the related research field on technostress, Riedl, 2012; La Torre et al., 2019, 2020).

Moreover, combining physiological measures with other data collection methods, such as implicit measures or behavioral observations (e.g., choice tasks or response time), could provide valuable insights into the complex interplay of cognitive, affective, and behavioral components that shape AI attitudes. Integrating these complementary methods would enable researchers to not only identify potential biases in self-report data but also to uncover the underlying psychological processes that drive individuals’ reactions to AI and related technologies.

Future research may benefit from exploring the impact of various factors on AI attitudes, such as personality traits, cognitive abilities, and exposure to AI technology. This would contribute to a more comprehensive understanding of the factors that influence AI attitudes and help identify potential intervention areas to enhance AI acceptance and adoption. Future studies might also investigate the relationship between AI attitudes and actual AI technology usage, examining whether 4-items AIAS scores predict technology adoption and user behavior. This would provide evidence of the scale’s criterion validity and practical utility for understanding and predicting AI technology adoption in real-world contexts.

The 4-items AIAS scale could also be employed to longitudinally evaluate changes in societal attitudes toward AI during periods of rapid development and implementation of AI technologies. This would provide an index to track the status of AI technology acceptability and contribute to a better understanding of the evolving public perception of AI.

While the AIAS has shown effectiveness in gaining insights into people’s attitudes toward AI, it presents a somewhat simplified and general perspective of these attitudes. AI, as a technology, is becoming more nuanced, sophisticated, and human-like in its abilities and characteristics. This advancement and complexity of AI technology inherently make people’s attitudes and perceptions toward it more complex.

For instance, the recent theoretical developments of AI’s self-prolongation and autonomous minds are elements that go beyond the bounds of what AIAS can directly assess. The expansion of AI’s roles in society and its increasing sophistication adds several layers of complexity that the human mind must challenge when thinking about AI agents. Recent research based on the information-processing-based Bayesian Mindsponge Framework (BMF, see “Mindsponge Theory,” Vuong, 2023) found that people’s perceptions of an AI’s autonomous mind were influenced by their beliefs about the AI’s desire for self-prolongation (Vuong et al., 2023). This suggests a directional pattern of value reinforcement in perceptions of AI and indicates that as AI becomes more sophisticated in the future, it will be harder to set clear boundaries about what it means to have an autonomous mind, therefore changing the perception, evaluation, and the core attitude toward AI agents.

The value of the AIAS primarily lies in its convenience and its ability to capture a snapshot of basic attitudes toward AI. However, its simplicity also implies a limitation in capturing the full complexity and range of attitudes toward increasingly human-like AI. Therefore, it is imperative to acknowledge this lack of complexity in the scale as a limitation of the current study.

## 5. Conclusion

In conclusion, the AIAS offers a valuable tool for researchers and practitioners to assess attitudes toward AI technology. The scale can be used to explore factors influencing AI acceptance and adoption, inform the development of AI applications that are better aligned with user needs and expectations, evaluate development of the perception of the AI while the technology and contribute to a more comprehensive understanding of the complex interplay between AI technology and society.

The 4-items AIAS scale that was validated in this article is available in pdf format in the supplementary material of the present article (CC BY 4.0) as well as on the open research data repository Zenodo. The file also contains instructions for scoring. The validated 4-items AIAS can be also be retrieved from the following DOI and used freely.^1^ Please refer to this article when using the scale.

## Data availability statement

The raw data supporting the conclusions of this article will be made available by the author, without undue reservation.

## Ethics statement

Ethical review and approval was not required for the study on human participants in accordance with the local legislation and institutional requirements. The patients/participants provided their written informed consent to participate in this study.

## Author contributions

SG was responsible for the development of the main survey, funding acquisition, the analysis, and article writing.

## Conflict of interest

The author declares that the research was conducted in the absence of any commercial or financial relationships that could be construed as a potential conflict of interest.

## Publisher’s note

All claims expressed in this article are solely those of the authors and do not necessarily represent those of their affiliated organizations, or those of the publisher, the editors and the reviewers. Any product that may be evaluated in this article, or claim that may be made by its manufacturer, is not guaranteed or endorsed by the publisher.

## Footnotes

^1^https://doi.org/10.5281/zenodo.7757534
