# Supplementary material for: Development and validation of the AI attitude scale (AIAS-4): a brief measure of general attitude toward artificial intelligence
Source: Front Psychol. 2023 Jul 24;14:1191628. doi: 10.3389/fpsyg.2023.1191628 (PMC10406504; doi:10.3389/fpsyg.2023.1191628)
Supplement: Supplementary file 1 [file Data_Sheet_1.PDF]

AI Attitude Scale (AIAS-4 Scale)

Below you will find sentences about the attitude toward Artificial Intelligence (AI)

1. **<u>I believe that AI will improve my life</u>**

Not at all 1 2 3 4 5 6 7 8 9 10 Completely agree

2. **<u>I believe that AI will improve my work</u>**

Not at all 1 2 3 4 5 6 7 8 9 10 Completely agree

3. **<u>I think I will use AI technology in the future</u>**

Not at all 1 2 3 4 5 6 7 8 9 10 Completely agree

4. **<u>I think AI technology is positive for humanity</u>**

Not at all 1 2 3 4 5 6 7 8 9 10 Completely agree

Instructions for scoring:

AIAS-4: Average of all the item scores
